# Clinicopathologic Analysis of Cathepsin B as a Prognostic Marker of Thyroid Cancer

**DOI:** 10.3390/ijms21249537

**Published:** 2020-12-15

**Authors:** Eun-Kyung Kim, Min-Jeong Song, Ho Hee Jang, Yoo Seung Chung

**Affiliations:** 1 Department of Biochemistry, College of Medicine, Gachon University, Incheon 21999, Korea; ekkim@gachon.ac.kr (E.-K.K.); neptune6nrg@hanmail.net (M.-J.S.); 2 Department of Surgery, Gil Medical Center, College of Medicine, Gachon University, Incheon 21999, Korea

**Keywords:** cathepsin b, thyroid cancer, epithelial mesenchymal transition, metastasis

## Abstract

Thyroid cancer incidence has increased worldwide; however, investigations of thyroid cancer-related factors as potential prognosis markers remain insufficient. Secreted proteins from the cancer secretome are regulators of several molecular mechanisms and are, thereby, ideal candidates for potential markers. We aimed to identify a specific factor for thyroid cancer by analyzing the secretome from normal thyroid cells, papillary thyroid cancer (PTC) cells, and anaplastic thyroid cancer cells using mass spectrometry (MS). Cathepsin B (CTSB) showed highest expression in PTC cells compared to other cell lines, and CTSB levels in tumor samples were higher than that seen in normal tissue. Further, among thyroid cancer patients, increased CTSB expression was related to higher risk of lymph node metastasis (LNM) and advanced N stage. Overexpression of CTSB in thyroid cancer cell lines activated cell migration by increasing the expression of vimentin and Snail, while its siRNA-mediated silencing inhibited cell migration by decreasing vimentin and Snail expression. Mechanistically, CTSB-associated enhanced cell migration and upregulation of vimentin and Snail occurred via increased phosphorylation of p38. As our results suggest that elevated CTSB in thyroid cancer induces the expression of metastatic proteins and thereby leads to LNM, CTSB may be a good and clinically relevant prognostic marker.

## 1. Introduction

Thyroid cancer is the most common endocrine-related cancer worldwide, and Surveillance, Epidemiology, and End Results Program data for the last 10 years (2009–2018) reveal that the incidence of thyroid cancer has been stable by about 3% of the new cancer cases. However, associated death rates have also been rising by 0.6%, on average, each year [1]. Thyroid cancer in Korea is the second most common in women and fourth most common cancer in general. Even though prognosis in thyroid cancer is excellent and the 5-year survival rate is 98%, the 5-year survival rate drops to 54.9–62.0% in advanced cases [2,3]. Thus, as thyroid cancer is rather common and the prognosis is unfavorable in advanced-stage cancer, identification of a useful molecular marker related to thyroid cancer is essential. BRAF, RAS, TP53, and PIK3CA mutations have been used for diagnosis or prognosis of follicular cell-driven thyroid neoplasm [4,5]. Recently, TERT mutation or apolipoprotein E have reported as a prognostic factor of papillary thyroid cancer (PTC) [6,7]. However, we thought that the searching for more diverse molecular markers and research methods were needed.

Proteomic analysis is routinely used to identify differentially expressed proteins (DEPs) as potential prognostic cancer markers [8,9,10]. iTRAQ-MS, which is a quantitative mass spectrometric method, has been previously used to reveal that, compared to normal thyroid tissue, papillary thyroid carcinomas exhibit loss of E-cadherin and differentiation markers [11]. Additionally, liquid chromatography–tandem mass spectrometry (LC–MS/MS) analysis of PTC cells has demonstrated alterations in the actin cytoskeleton [12]. Despite this, thyroid cancer-related markers are scarce and investigations of their mechanism(s) of action in thyroid cancer progression are currently poorly understood.

Many cancers generate secretome for the regulation of microenvironments through matrix remodeling, alternation of the cell to cell interaction, and angiogenesis, and because these secreted molecules act as either autocrine or paracrine factors, the secretome has an important function in cancer progression [13,14,15]. The secretome consists of metabolites and several proteins such as cytokines, hormones, and growth factors [13,16]. Therefore, it is useful to investigate secreted proteins and their mechanisms of action in cancer progression and metastasis because they are potential cancer regulators.

Cathepsin B (CTSB), a lysosomal cysteine protease, is a member of the cysteine protease family, and experimental models have shown that it has a central role in multiple pathologic processes, including initiation, tumor cell proliferation, growth, angiogenesis, and metastasis [17,18]. CTSB is also associated with metalloproteinase regulation, intracellular communication, autophagy induction, immune resistance, and cell survival [19]. High expression levels of CTSB have been observed in various cancers such as breast, esophageal, gastric, colon, and pancreatic cancers, and hepatocellular carcinoma, and both advanced stage and poor survival in multiple cancers are known to be associated with greater expression of CTSB [20,21,22,23].

The relationship between CTSB and thyroid cancer is not known, and this study investigated differential CTSB expression, if any, in the secretome of a PTC cell line and compared it with that seen in a normal thyroid cell line. Changes in CTSB expression were validated using PTC surgical specimens, which also revealed a CTSB-dependent mechanism of metastasis.

## 2. Results

### 2.1. CTSB Is Highly Expressed in Papillary Cancer

**Figure 1:**
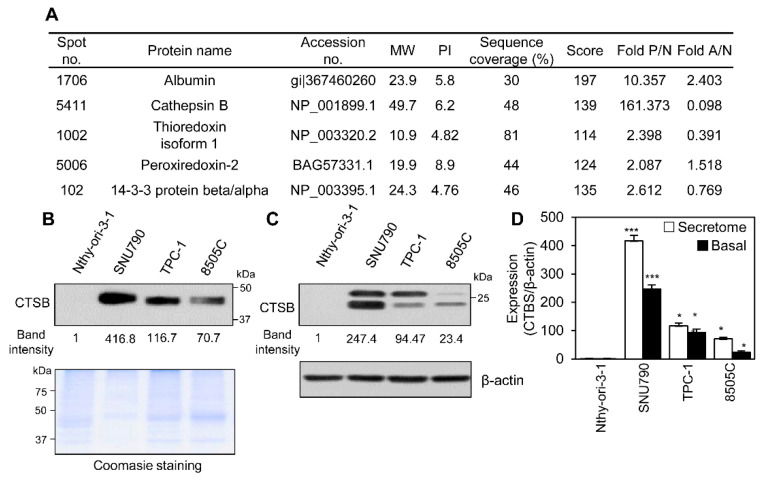
Higher expression of cathepsin B (CTSB) in PTC cell lines. (**A**) The identified list of DEPs in the secretome of thyroid epithelial cell (N), PTC cell (P), and anaplastic thyroid cancer cell (A) using proteomic analysis. (**B**) Western blot of secreted CTSB levels in conditioned media of cultured thyroid cancer cell lines (top). Proteins of the secretome were stained with CBB-R250 on the SDS–PAGE gel (bottom). (**C**) Analysis of basal CTSB levels in Nthy-ori-3-1, SNU790, TPC-1, and 8505C cells using Western blotting. (**D**) Analysis of relative CTSB expression in the secretome and its basal levels in thyroid cancer cell lines. The band intensity of CTSB expression was measured with ImageJ software and was normalized to β-actin or Coomassie staining. The relative intensity of CTSB in the Nthy-ori-3-1 was set to 1. Band intensity of CTSB level in SNU790, TPC-1, and 8505C was reported as fold-change compared to Nthy-ori-3-1. * *p* < 0.05, *** *p* < 0.001.

We hypothesized that the thyroid cancer secretome contains cancer-related factors and, therefore, collected conditioned media from epithelial (N), papillary (P), and anaplastic (A) thyroid cancer cell types to identify potentially useful prognostic factors. We used the Nthy-ori-3-1 cell line for normal thyroid epithelial cells, the SNU 790 cell line for PTC cells, and 8505C cell line for anaplastic thyroid cancer cells. Conditioned media were subjected to two-dimensional polyacrylamide gel electrophoresis (2D-PAGE) to identify DEPs and spots with differential intensities were compared to normalized spot intensity of the secretome of epithelial thyroid cell. Proteins were subsequently identified by mass spectrometry (MS). Analysis of differences in relative expression of the DEPs between papillary and epithelial or anaplastic types (Figure 1A and Figure S1) showed that cathepsin B (CTSB) expression was about 161-times higher in PTC cells compared to epithelial thyroid cells.

CTSB is produced in the form of a precursor (pro-CTSB) of about 44 kDa and undergoes proteolytic processing and glycosylation to become a mature form containing a heavy chain (running at 27 and 24 kDa) and a light chain (5 kDa). It is known that intracellular CTSB is mainly found in mature form, and extracellular CTSB is mainly found in precursor form [24,25]. We quantified secreted CTSB levels in conditioned media using Western blotting. TPC-1 cells are a PTC cell line. Secreted CTSB was higher in SNU790 and TPC-1 cells; however, it was absent or seen in low levels in conditioned media from Nthy-ori-3-1 cells (Figure 1B). Therefore, we identified CTSB as a potential key regulator of PTC and hypothesized that changes in CTSB expression affect thyroid cancer progression.

The cancer secretome may originate from cancer cells themselves [14], and to evaluate if the observed high expression of CTSB originated in the cells themselves, we measured CTSB levels in Nthy-ori-3-1, SNU790, TPC-1, and 8505C cell lines using immunoblotting (Figure 1C). CTSB was not detected in normal Nthy-ori-3-1 cells, and this coincided with low expression seen in the secretome of Nthy-ori-3-1 cells. In contrast, SNU790 and TPC-1 cell lines showed higher expression of CTSB, and 8505C cells showed lower expression of cellular basal CTSB compared to SUN790 and TPC-1 cells. Expression intensity analysis using ImageJ showed a similar pattern of CTSB expression in the cells and in the secretome (Figure 1D). These results suggest that overexpression of CTSB in the secretome originates in the cells and that its higher expression is specific to PTC cells.

### 2.2. Elevated CTSB Is Linked to Lymph Node Metastasis in Clinical Samples

**Figure 2:**
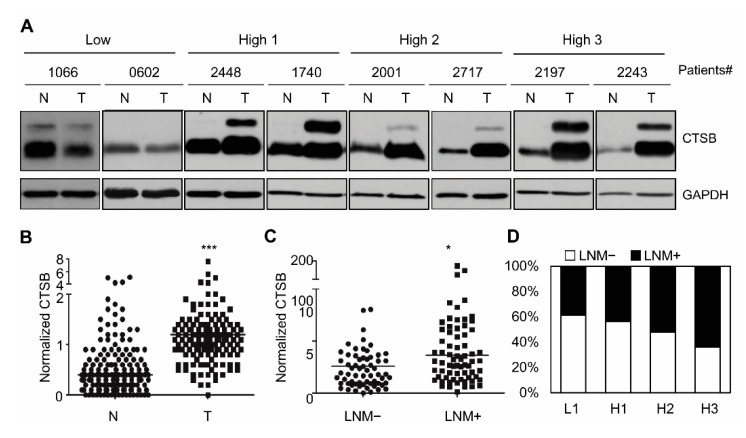
Higher expression of CTSB in PTC in clinical samples. (**A**) Expression of CTSB in normal (N) and tumor (T) samples from thyroid cancer patients. The band intensity of CTSB expression was measured with ImageJ and was normalized to GAPDH. The relative intensity of CTSB in normal tissue was set to 1. The band intensity of CTSB level in tumor tissue was calculated as fold-change compared to normal tissue. (**B**) Analysis of CTSB level between normal (N) and tumor (T) tissues in the patient samples of thyroid cancer. CTSB band intensity was normalized to GAPDH and was quantified using ImageJ. *** *p* < 0.001 (**C**) Analysis of CTSB expression in clinical samples with or without LNM. * *p* < 0.05 (**D**) Analysis of the relationship between occurrence (%) of LNM and CTSB expression levels.

To explore the relationship between the CTSB expression level and clinicopathologic features in thyroid cancer, we analyzed the expression levels of CTSB in 158 pairs of Korean PTC patients (tumor tissue vs. adjacent nontumor tissue) using Western blotting. Clinicopathological characteristics of the cohort, such as gender, age, tumor size, extrathyroidal extension, lymphovascular invasion, lymph node metastasis (LNM), and TNM stage, are listed in Table 1. Table 1 shows the relationship of CTSB expression and prognostic factors of PTC. Tumor size was larger in High 3 group than low group. Multiplicity was significantly higher in accordance with increasing of expression of CTSB. High expression of CTSB affected LNM and N stage. Immunoblotting of patient samples showed higher CTSB levels in tumor tissue compared to normal tissue (Figure 2A,B), and patients with higher CTSB expression were at increased risk of LNM and advanced N stage (Figure 2C,D). These results indicate that elevated CTSB expression in thyroid cancer is probably related to metastasis in clinical settings.

### 2.3. Changes in CTSB Expression Regulate Metastasis in Thyroid Cancer Cells

**Figure 3:**
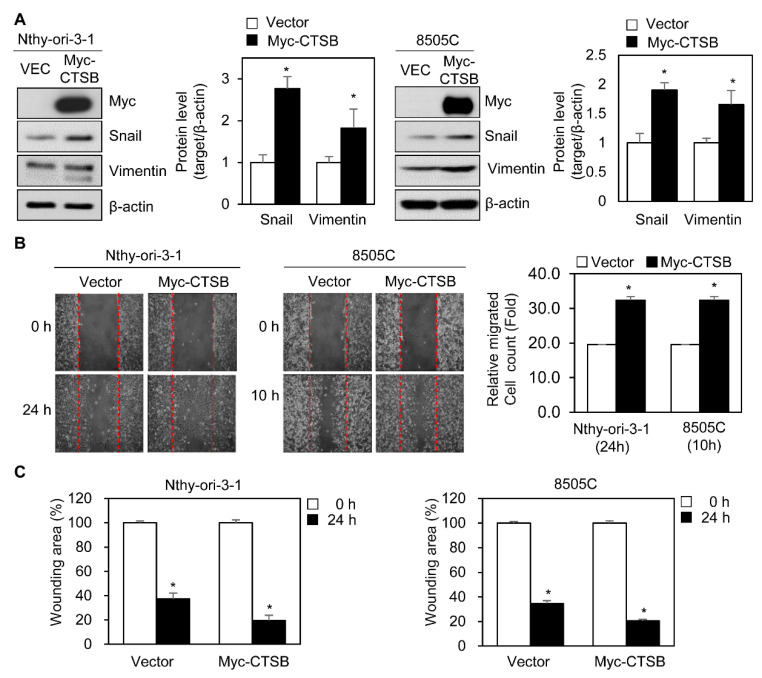
Overexpression of CTSB expression induces activation of cell migration in normal thyroid epithelial and thyroid cancer cell lines. (**A**) Immunoblotting for expression levels of Snail and vimentin in Nthy-ori-3-1 and 8505C cells transfected with plasmids for myc-CTSB or vector for 24 h. The table for the relative levels of Snail or vimentin with overexpression of CTSB. The band intensity of Snail or vimentin was determined using ImageJ program and normalized to that of the band intensity of ꞵ-actin. The relative level of Snail or vimentin was calculated and statistically analyzed. (**B**) Scratch wound healing assay in Nthy-ori-3-1 and 8505C cells transfected with myc-tagged CTSB or vector for 24 h. (**C**) The wounding area (%) of Nthy-ori-3-1 or 8505C cells with the overexpressed CTSB for 24 h. Changes in relative migrating cell numbers and wounding area were captured by microscopy and counted using ImageJ. Data are representative of three independent experiments. * *p* < 0.05.

Analysis of patient samples showed that upregulation of CTSB was associated with LNM (Figure 2C,D); therefore, we hypothesized that changes in CTSB expression can affect metastasis in thyroid cancer. To investigate this, we designed cloning of the human CTSB gene into plasmid pCS4-3xMyc and obtained pCS4-3xMyc-CTSB (Myc-CTSB). We transfected a plasmid carrying Myc-CTSB to cell lines that show low levels of basal CTSB. Snail and vimentin were used as representative markers of mesenchymal transition [26]. CTSB overexpression induced an increase in Snail and vimentin levels in Nthy-ori-3-1 and 8505C cells (Figure 3A), apart from enhancing the number of migrating cells, compared to the vector group, in the scratch assay (Figure 3B). Analysis of wounding area (%) showed overexpression of CTSB filled faster the scratch area compared to the vector group (Figure 3C).

**Figure 4:**
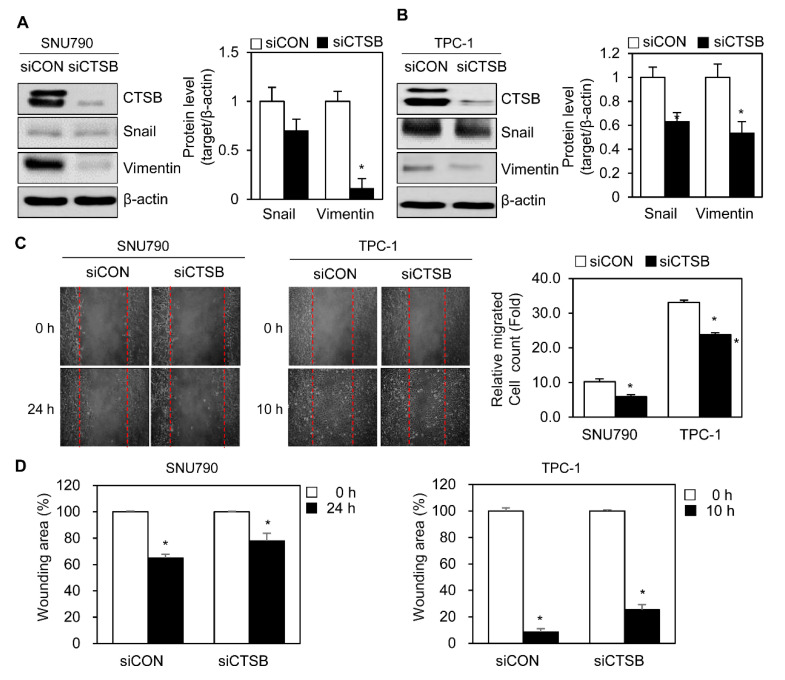
Knock down of CTSB expression inhibits activation of cell migration in PTC cell lines. (**A**,**B**) Immunoblotting for CTSB, Snail, and vimentin after transfection with siCON or siCTSB in SNU790 and TPC-1 cells for 24 h. Table for the relative levels of Snail or vimentin in the knock down of CTSB. The band intensity of Snail or vimentin was calculated using ImageJ program and normalized to that of the band intensity of β-actin. The relative level of Snail or vimentin was calculated and statistically analyzed. (**C**) Wound healing assay in SNU790 and TPC-1 after transfection with siCON or siCTSB. (**D**) The wounding area (%) of SNU790 (24 h) or TPC-1 (10 h) cells with the treatment of siCON or siCTSB. The number of the relative migrating cells or wounding area was analyzed using ImageJ in SUN790 cells at 24 h and in TPC-1 cells at 10 h. Band intensity was normalized to β-actin expression and quantified using ImageJ. Data are representative of three independent experiments. * *p* < 0.05.

Next, to determine whether loss of basal CTSB reduced metastasis, SNU790, and TPC-1 cells, which constitutively express high levels of CTSB, were treated with siRNA against CTSB. Inhibited expression of CTSB led to reduced levels of Snail and vimentin in SNU-790 and TPC-1 cells (Figure 4A,B), apart from lowering migrating cell numbers in the scratch assay (Figure 4C). Analysis of wounding area (%) showed that knock down of CTSB levels remained more in the wound area compared to the siCON group (Figure 4D).

Taken together, these results indicate that altering cellular levels of CTSB can affect epithelial–mesenchymal transition (EMT) activation in thyroid cancer cell lines.

### 2.4. Secreted CTSB Induces Activation of EMT via Intercellular Signaling in Thyroid Cancer Cells

**Figure 5:**
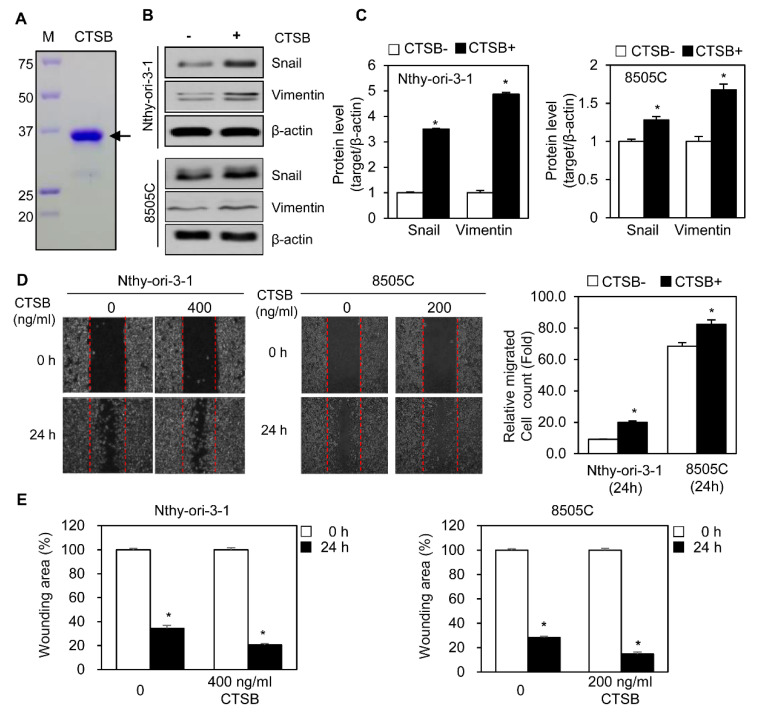
Treatment with recombinant CTSB induces cell migration in normal thyroid epithelial and thyroid cancer cell line. (**A**) Recombinant CTSB was produced in an *Escherichia coli* (*E. coli*) system. Purified CTSB was subjected to SDS–PAGE and stained by Coomassie blue to confirm the purity of CTSB. Arrow indicates purified CTSB protein band. (**B**) Western blotting for Snail and vimentin in Nthy-ori-3-1 and 8505C cells after treatment with recombinant CTSB. Nthy-ori-3-1 cells were treated with 400 ng/mL CTSB for 24 h while 8505C cells were treated with 200 ng/mL CTSB for 24 h. (**C**) Tables for the relative levels of Snail or vimentin exposure to recombinant human CTSB. The band intensity of Snail or vimentin was determined using ImageJ program and normalized to that of the band intensity of β-actin. The relative level of Snail or vimentin was calculated and statistically analyzed. (**D**) Scratch wound healing assay in Nthy-ori-3-1 and 8505C cells after treatment with recombinant CTSB for 24 h. Pictures were captured on an inverted microscope. Cells were treated with 400 or 200 ng/mL recombinant CTSB for 24 h. (**E**) The wounding area (%) of Nthy-ori-3-1 (400 ng/mL) or 8505C (200 ng/mL) cells with the treatment of siCON or siCTSB. The calculation of migrating cells or wounding area was counted by ImageJ. Data are representative of three independent experiments. * *p* < 0.05.

To assess whether secreted CTSB affects EMT progression, we evaluated changes in EMT markers after recombinant CTSB treatment of cell lines that express low levels of CTSB. Recombinant human CTSB was expressed and purified from an *Escherichia coli* (*E. coli*) system (Figure 5A). Nthy-ori-3-1 and 8505C cells were treated with recombinant CTBS at different concentrations, followed by cell migration assay to select an appropriate concentration. Nthy-ori-3-1 cells were treated with recombinant CTSB (400 ng/mL) for 24 h. 8505C cells were treated with recombinant CTSB (200 ng/mL) for 24 h. Immunoblotting showed that the treatment with recombinant CTSB increased Snail and vimentin levels in Nthy-ori-3-1 and 8505C cells (Figure 5B,C). To explore the physiological effect of secreted CTSB, we used the wound healing assay in Nthy-ori-3-1 and 8505C cell lines treated with recombinant CTSB and found greater numbers of migrating cells in treated cells compared to those that were not treated (Figure 5D). Analysis of wounding area (%) showed treatment of recombinant CTSB induced more closure of the wound area than nontreatment in Nthy-ori-3-1 and 8505C for 24 h (Figure 5E). These results indicate that secreted CTSB can accelerate cell migration in thyroid cancer cell lines.

**Figure 6:**
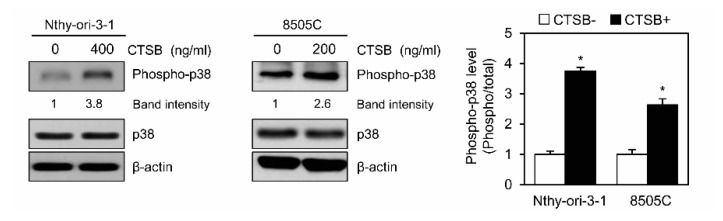
Treatment with recombinant CTSB induces p38 activation in normal thyroid epithelial and thyroid cancer cell lines. Phosphorylation of p38 was ascertained by immunoblotting. Nthy-ori-3-1 cells were treated with 400 ng/mL CTSB for 1 h while 8505C cells were treated with 200 ng/mL CTSB for 1 h. Intensity of phosphor-p38 bands were normalized to total p38 levels and were quantified using ImageJ software. The relative intensity of phosphor-p38 without CTSB was set to 1. Band intensity of phospho-p38 with the treatment of CTSB was analyzed as fold-change compared to no treatment of CTSB. Table for the relative levels of phosphor-p38 exposure to recombinant human CTSB. The relative level of phosphor-p38 was calculated and statistically analyzed. Data are representative of three experiments. * *p* < 0.05.

To explore the mechanism underlying the effects of secreted CTSB during EMT progression, we analyzed changes in cellular signaling pathways in CTSB-treated thyroid cancer cell lines. Nthy-ori-3-1 or 8505C cells were treated with recombinant CTSB at a concentration of 400 or 200 ng/mL, respectively, for 1 h, and then the cells were harvested. Cell lyastes were subjected to immunoblotting to detect the signaling molecules. The results showed enhanced p38 phosphorylation upon exposure to CTSB in these cell lines (Figure 6), suggesting that secreted CTSB can affect intercellular signaling to promote cell migration.

## 3. Discussion

The most common endocrine cancer is thyroid cancer, and even as the thyroid cancer patients have steadily occurred, their survival rate has also reduced in the last 10 years [1,2]. Thyroid cancer is the first most common cancer among men and women of the 15–34 age group in Korea and there is an urgent need for an effective diagnostic and prognostic biological marker [3].

Thus far, there is scant research on CTSB and thyroid cancer. CTSB functions include contributions to thyroglobulin process and secretion of thyroxine from thyroid cells [27,28,29]. In contrast, CTSB in PTC is localized to the basement membrane, where it induces EMT by changing the properties of the extracellular matrix [30]. Even though previous studies have reported on the level and localization of CTSB in thyroid cancer, the underlying mechanisms of action remain unknown. Here we reveal this mechanism to be a metastasis process via p38 activation and also show that it is regulated by CTSB secreted from thyroid cancer cells.

Overexpression or abnormal activity of CTSB has been previously reported in breast, pancreatic, liver, colorectal, oral, and lung cancer, and among others [22,23,25,30,31,32]. Higher levels of CTSB have been demonstrated in neoplastic thyroid disease compared to non-neoplastic disease [33]. Our data show that real-world patient samples of PTC also express higher levels of CTSB and that the enhanced levels were related to LNM. These observations offer a new strategy for determining clinical prognosis after LNM in PTC. Cathepsin S, another protein of cysteine protease family, was reported to be related with more frequent LNM and advanced tumor-node-metastasis stages compared with the low-expression group. The authors suggested that Cathepsin S might be used as a predictive marker for the prognosis of PTC [34].

The mechanisms of CTSB action in thyroid cancer are unknown and our investigation of the same revealed that CTSB overexpression induces cell migration by enhancing the expression of vimentin and Snail in thyroid cancer cell lines. Previous studies have reported that siRNA-induced downregulation of CTSB inhibited EMT in other cancer cell lines [25,35]. We similarly demonstrate that low levels of CTSB inhibit cell migration through decreased expression of vimentin or Snail in thyroid cancer cell lines. Thus, it appears that CTSB exerts its effects during metastasis by regulating Snail and vimentin.

While it is known that secreted CTSB significantly alters the tumor microenvironment during invasion, its mechanism of action and associated intracellular signaling pathway(s) have not yet been determined [30]. Our investigations on the role of secreted CTSB during thyroid cancer metastasis show activation of the p38 pathways without any involvement of the ERK and JNK pathways (data not shown) or cell cycle-related factors (data not shown). Thus, even though secreted CTSB is located on the extracellular membrane, the identity of cognate receptors for signal transduction remains unknown.

## 4. Materials and Methods

### 4.1. Reagents and Antibodies

All chemicals used in this investigation were purchased from Sigma (Sigma, St. Louis, MO, USA) and were of electrophoresis or analytical grade. Chemicals used were 4-Sulfophenyl isothiocyanate, urea, thiourea, iodoacetamide, α-cyano-4-hydroxycinnamic acid (CHCA), acetonitrile, Bradford solution, SDS, benzamidine, bis-acrylamide, ammonium bicarbonate, trifluoroacetic acid, dithiothreitol (DTT), acrylamide, 3-((3-cholamidopropyl) dimethyammonio)-1-propanesulfonate (CHAPS), and sodium bicarbonate. The solution of pharmalyte (pH 3.5–10) was purchased from Amersham Biosciences (Amersham Biosciences, Piscataway, NJ, USA). Sequencing-grade modified porcine trypsin was obtained from Promega (Madison, WI, USA). Antibodies for CTSB (#31718), Snail (#3879), Vimentin (#5741), phospho-p38 (#4511), p38 (#8690), β-actin (#4970), and horseradish peroxidase conjugated secondary antibodies (#7074 and #7076) were purchased from Cell Signaling Technology (CST, Danvers, MA, USA).

### 4.2. Cell Culture

The following cell lines were used. Human PTC cell lines were SNU790, TPC-1, 8505C, while normal thyroid epithelial cell line was Nthy-ori-3-1. SNU790 cell line was purchased from the Korean Cell Line Bank (KCLB, Seoul, Korea). TPC-1, 8505C, and Nthy-ori-3-1 cell lines were purchased from Sigma-Aldrich (Sigma, St. Louis, MO, USA). These cell lines were cultured in RPMI 1640 supplemented with 10% fetal bovine serum (FBS), 100 μg/mL streptomycin, and 100 U/mL penicillin (CAPRICORN, Germany). Cells were cultured in an incubator at 37 °C with 5% CO_2_.

### 4.3. Protein Preparation for MS Analysis

Cells were grown for 48 h under serum free conditions, 20 mL of culture medium was collected and centrifuged at 1000× *g* for 10 min to remove cell debris. The supernatants were harvested, filtered using a syringe membrane filter with a 0.22 um pore (Millipore, Billerica, MA, USA), and then secreted proteins were concentrated using centrifugal filter units with a 3-kDA cutoff at 4  °C (Amicon Ultra Centrifugal filters, Millipore, St. Louis, Mo, USA). 2DE-lysis solution (2 M thiourea containing 4% (*w*/*v*) 3-((3-cholamidopropy) dimethyammonio)-1-propanesulfonate (CHAPS), 7 M urea, 1 mM benzamidine, 1% (*w*/*v*) dithiothreitol (DTT), and 2% (*v*/*v*) pharmalyte) was added to 150 µL of concentrated supernatant by incubating it for 30 min at room temperature with vortexing. After centrifugation at 15,000× g for 1 h at 4 °C, the insoluble pellet was discarded, and the soluble sample was performed for 2D-PAGE. Protein quantification was estimated using 2D Quantitative kit (Amersham Biosciences, Piscataway, NJ, USA).

### 4.4. Two-Dimensional PAGE and Image Analysis

To equilibrate IPG strips, dry strips (4–10 NL IPG, 24 cm, Genomine, Pohang, Korea) were soaked for 12–16 h in the pre-equilibration solution (2 M thiourea with 2% 3-((3-cholamidopropy) dimethyammonio)-1-propanesulfonate (CHAPS), 7 M urea, 1% (*w*/*v*) pharmalyte, and 1% (*w*/*v*) DTT), and loaded with 200 µg (150 µL) of protein per strip. Isoelectric focusing (IEF) was conducted at 20 °C using a Multiphor II electrophoresis unit with EPS 3500 XL power supply (Amersham Biosciences, Piscataway, NJ, USA) according to manufacturer’s instructions. IEF voltage was linearly raised from 150 to 3500 V for 3 h to enable sample entry and maintained at 3500 V. Focusing was considered complete by 96 kVh. Before electrophoresis in the second dimension, the strip was sequentially incubated for 10 min each in two equilibration buffers (50 mM Tris-Cl (pH 6.8) with 6 M urea, 2% (*w*/*v*) SDS, and 30% (*w*/*v*) glycerol) such that the first buffer contained 1% (*w*/*v*) DTT and the second had 2.5% (*w*/*v*) iodoacetamide. Equilibrated strips were embedded onto SDS–PAGE gels (20 × 24 cm, 10–16%) and run on a Hoefer DALT 2D system (Amersham Biosciences, Piscataway, NJ, USA) according to manufacturer’s instructions. SDS–PAGE was performed at 20 °C for 1700 Vh and then silver stained.

Digitized images were quantitatively analyzed using PDQuest (version 7.0, BioRad, Hercules, CA, USA) software and the unit of peptide spots was normalized based on the intensity of the total number of effective spots. To obtain physiologically meaningful results, peptide spots were chosen such that the variation in protein staining intensity was at least more than two-fold compared to normal samples.

### 4.5. MS

To identify protein spots by peptide mass fingerprinting, protein spots were digested using trypsin, mixed with α-cyano-4-hydroxycinnamic acid (CHCA) in 50% (*v*/*v*) 1% acetonitrile/0.1% TFA, and loaded on to a Microflex LRF 20 MALDI-TOF machine for analysis (Bruker Daltonics, Billerica, USA)

Spectra were obtained at 300 shots per spectrum over a m/z range of 600–3000 and were related to two-point internal calibration by trypsin auto-digestion peaks (m/z 842.5099, 2211.1046). A list of peaks was compiled using Flex Analysis, ver. 3.0 (Bruker Daltonics, Billerica, USA). The thresholds used for peak identification were minimum resolution of a monoisotopic mass of 500 and a S/N value of 5. MASCOT, which is a search program and developed by Matrixscience (http://www.matrixscience.com/), was utilized to identify proteins based on peptide mass fingerprinting profiles and the following criteria were used in the database search: (i) the digestion by trypsin, (ii) up to one lost cleavage, (iii) full modification by 2-iodoacetamide (Cys), (iv) oxidation (Met) as partial modification, (v) monoisotopic masses, and (vi) a mass tolerance of ±0.1 Da. PMF acceptance criteria were also used in probability score calculation.

### 4.6. TCA Precipitation and SDS–PAGE Staining

Cells were cultured in 10 mL of medium with 100 μg/mL streptomycin, and 100 U/mL penicillin (Capricorn Scientific, GmbH, Ebsdorfergrund, Germany) without FBS for 48 h at 37 °C with 5% CO_2_. To remove suspended debris, harvested cultured medium was centrifuged at 4000 rpm for 5 min at 4 °C. TCA solution was added to the harvested medium to a final concentration of 12% TCA, vortexed, and centrifuged at 12,000 rpm for 15 min at 4 °C. Supernatants were discarded. The precipitated pellets were suspended by adding 1 mL of 100% acetone and centrifuged at 12,000 rpm for 7 min at 4 °C. This process was repeated three times and air dried at room temperature for 5 min. Dried pellets were dissolved in a solution containing 1% SDS, 100 mM Tris-HCl (pH 8.0), and 1 mM EDTA at 25 °C for 15 min, samples mixed with loading buffer, boiled at 100 °C for 5 min, and subjected to SDS–PAGE. Separated proteins were transferred onto nitrocellulose membranes (NC membrane; GE Healthcare) and subjected to Western blotting, while the PAGE gel was stained with Coomassie Blue R-250 dye after electrotransfer.

### 4.7. Western Blotting Analysis

Collected cells were lysed in RIPA buffer (0.5% sodium deoxycholate, 0.1% SDS, 150 mM NaCl, 50 mM Tris (pH 8.0), and 1% NP-40) containing a protease inhibitor cocktail (GenDEPOT, Barker, TX, USA). About 30 μg of cell lysate was mixed with reducing loading buffer, boiled at 100 °C for 5 min, separated by SDS–PAGE, and electrotransferred onto nitrocellulose membranes (NC membrane; GE Healthcare). Transferred NC membranes were soaked in TBST (Tris-buffered saline with 0.1% Tween 20) containing 8% non-fat milk for 30 min at room temperature to block nonspecific binding sites. Blots were incubated with primary antibodies (CTSB at 1:1000, GAPDH at 1:1000, Myc at 1:1000, Snail at 1:1000, Vimentin at 1:1000, and β-actin at 1:1000) either overnight at 4 °C, washed three times with TBST, incubated with horseradish peroxidase (HRP)-conjugated secondary antibodies (1:3000) for 2 h at room temperature, washed thrice with TBST, and developed using ECL reagents (Amersham, Piscataway, NJ, USA).

### 4.8. Biopsy Samples

Biopsy samples of patients with PTC were retrospectively collected and analyzed. A total of 158 patients were diagnosed and underwent surgery at the Gachon University Gil Medical Center in Korea between September and December 2017. Written informed consent was obtained from all participants. Patients with recurrence of PTC were excluded. Clinicopathologic prognostic factors related to PTC were analyzed based on data collected from medical, pathologic, and surgical records. Thyroid cancers were staged according to criteria issued by the American Joint Committee on Cancer classification (AJCC, 8th edition). This study was approved by the Institutional Ethical Committee and Review Board of the Gachon University Gil Medical Center (12 May 2015, IRB No. GAIRB2015-122).

### 4.9. Analysis of CTSB Level Using Patient Sample

The tissue of normal or tumor was lysed in RIPA buffer with metal bead. About 30 μg of tissue lysate was mixed with loading buffer, separated by 10% SDS–PAGE and followed Western blotting procedure. The band intensities of CTSB expression were quantified by densitometric analysis using ImageJ software (National Institutes of Health) and normalized with the intensity of the GAPDH band in the same patient. The relative intensity of CTSB in normal tissue was set to 1. The band intensity of CTSB level in tumor tissue was calculated as fold-change compared to normal tissue. The expression levels of CTSB of tumor compared to normal tissue were classified into four groups according to band intensity. Low expression group was <1.5, High 1 group was ≥1.5 and <2, High 2 group ≥2 and <5, and High 3 group was ≥5. PTC patients’ clinicopathologic characteristics associated with prognosis were analyzed according to the degree of expression of CTSB.

### 4.10. Cell Transfection

CTSB was overexpressed in Nthy-ori-3-1 and 8505C cells cultured in 3 mL media of 6-well plates at a density of 4 × 10^5^ cells per well. After 16 h, 2 μg of pCS4-3xMyc-CTSB plasmid was transfected using Lipofectamine2000 (Invitrogen, CA) according to manufacturer’s guidelines. CTSB expression was knocked down using siRNA in SNU790 and TPC-1 cells. Both siCTSB (sc-29238) and a scrambled control siRNA (sc-37007) were obtained from Santa Cruz Biotechnology Inc (Santa Cruz, CA, USA). Cells were seeded in 6-well plates at a density of 4 × 10^5^ cells per well. After 16 h, transfection was performed using the TransIT-X2^®^ Transfection kit (Mirus, MIR6000, Madison, WI, USA), according to manufacturer’s instructions. After 48 h, transfected cells of plasmid or siRNA were harvested.

### 4.11. Wound Healing and Cell Counting

Cells were seeded at a density of 4 × 10^5^ cells/well in 6-well plates. At 80% confluence and at around 48 h post-transfection, wounds were created on the cell layers using the migration chamber (Cat.201903, SPL, Gyeonggi-do, Korea). Cells were gently washed with 1 mL of PBS to remove debris and suspended cells and the wound was monitored using a camera attached to an inverted optical microscope (×40) (Nikon, Tokyo, Japan). Relative migrated cell numbers in wound healing experiments were calculated using ImageJ software (NIH) [36]. Analysis of the area in wound healing experiments was calculated using MRI wound healing tool macro for ImageJ software (NIH)), (http://dev.mri.cnrs.fr/projects/imagejmacros/wiki/Wound_Healing_Tool). Relative wound closure was calculated by the method of calculation of percent wound confluence, as described previously [37,38].

### 4.12. Expression and Purification of CTSB

Recombinant human CTSB was expressed in *E. coli* Origami B (*DE3*) by transforming the plasmid pET21a-cDsbC-CTSB using standard protocols. Transformed *E. coli* were grown in Terrific broth (TB) at 37 °C and 200 rpm, along with antibiotics (50 μg/mL ampicillin, and 50 μg/mL kanamycin), till an optical density (OD_600_) of 0.7. Protein expression was induced by treatment with 0.1 mM IPTG at 20 °C for 18 h. Harvested *E. coli* were resuspended in PBS (pH 7.4), disrupted with sonication (5 s on/5 s off, total 7 min), centrifuged at 8000 rpm for 10 min at 4 °C, the soluble sample loaded onto a Ni-NTA column for separation, and washed with PBS. The recombinant CTSB protein was eluted with 200 mM imidazole and dialyzed in PBS, and the concentration of the purified recombinant CTSB was measured using the Nanodrop ND-1000 Spectrophotometer (Thermo Fisher, Waltham, MA, USA). Purity of recombinant CTSB was confirmed by subjecting 3 µg of protein to SDS–PAGE and staining with Coomassie Blue R-250 dye.

### 4.13. Statistical Analysis

All experiments were conducted in triplicate, and data are provided as mean ± standard deviation (SD). Comparisons between groups were made using the Pearson’s χ2 test, Fisher’s exact test, linear by linear associations, Student’s *t*-test, and ANOVA test to evaluate the significance of CTSB expression. Differences were deemed statistically significant at *p* < 0.05.

## 5. Conclusions

In summary, we show that changes in the expression or secretion of CTSB during PTC regulates metastasis via activation of p38-mediated EMT processes. Further studies are needed to identify receptors related to secreted CTSB signaling. Our results reveal that CTSB may not only be a potential prognostic marker of metastasis but also function as a new target for antimetastatic cancer drugs in thyroid cancer.

## Figures and Tables

**Table 1:** Clinicopathologic features of thyroid cancer patients.

Variables	Cathepsin B Expression				*p* Value	* *p* Value
	Low (n = 44)	High 1 (n = 16)	High 2 (n = 48)	High 3 (n = 50)		
**Gender**						
**Female**	35 (79.5%)	12 (75.0%)	37 (77.1%)	38 (76.0%)	0.978	n.s
Male	9 (20.5%)	4 (25.0%)	11 (22.9%)	12 (24.0%)		
**Age**	50.25 ± 12.77	50.94 ± 8.91	49.13 ± 11.10	44.74 ± 13.37	0.099	
**Size**	1.25 ± 0.80	1.77 ± 1.30	1.35 ± 0.89	1.80 ± 1.20	^#^ 0.031	
**ETE**						
No	15 (34.1%)	3 (18.8%)	16 (33.3%)	16 (32.0%)	0.719	n.s
Yes	29 (65.9%)	13 (81.3%)	32 (66.7%)	34 (68.0%)		
**LVI**						
No	34 (77.3%)	10 (62.5%)	32 (66.7%)	35 (70.0%)	0.621	n.s
Yes	10 (22.7%)	6 (37.5%)	16 (33.3%)	15 (30.0%)		
**LNM**						
No	27 (61.4%)	9 (56.3%)	23 (47.9%)	18 (36.0%)	0.058	0.007
Yes	17 (38.6%)	7 (43.8%)	25 (52.1%)	32 (64.0%)		
**T stage**						
1	33 (75.0%)	9 (56.3%)	36 (75.0%)	29 (58.0%)	0.297	n.s
2	6 (13.6%)	1 (6.3%)	4 (8.3%)	9 (18.0%)		
3	3 (6.8%)	4 (25.0%)	6 (12.5%)	10 (20.0%)		
4	2 (4.5%)	2 (12.5%)	2 (4.2%)	2 (4.0%)		
**N stage**						
0	27 (61.4%)	9 (56.3%)	23 (47.9%)	18 (36.0%)	0.053	0.049
1a	12 (27.3%)	6 (37.5%)	24 (50.0%)	25 (50.0%)		
1b	5 (11.4%)	1 (6.3%)	1 (2.1%)	7 (14.0%)		
**M stage**						
0	43 (97.7%)	16 (100.0%)	48 (100.0%)	50 (100.0%)	0.380	n.s
1	1 (2.3%)	0 (0.0%)	0 (0.0%)	0 (0.0%)		
**Stage**						
1	39 (88.6%)	14 (87.5%)	37 (77.1%)	42 (84.0%)	0.625	n.s
2	3 (6.8%)	2 (12.5%)	10 (20.8%)	7 (14.0%)		
3	1 (2.3%)	0 (0.0%)	1 (2.1%)	1 (2.0%)		
4	1 (2.3%)	0 (0.0%)	0 (0.0%)	0 (0.0%)		

ETE: extrathyroidal extension; LVI: lymphovascular invasion; LNM: lymph node metastasis. * *p* value: linear by linear association ^#^ significant between Low and High 3 in post-hoc Turkey’s HDS.

## Supplementary Materials

The following are available online at https://www.mdpi.com/1422-0067/21/24/9537/s1.

## Abbreviations

CTSB	Cathepsin B
DEP	Differentially expressed proteins
EMT	Epithelial–mesenchymal transition
LNM	Lymph node metastasis
MS	Mass spectrometry
PTC	Papillary thyroid cancer
