# Supplementary material for: Clinicopathologic Analysis of Cathepsin B as a Prognostic Marker of Thyroid Cancer
Source: Int J Mol Sci. 2020 Dec 15;21(24):9537. doi: 10.3390/ijms21249537 (PMC7765333; doi:10.3390/ijms21249537)
Supplement: Supplementary file 1 [file ijms-21-09537-s001.pdf]

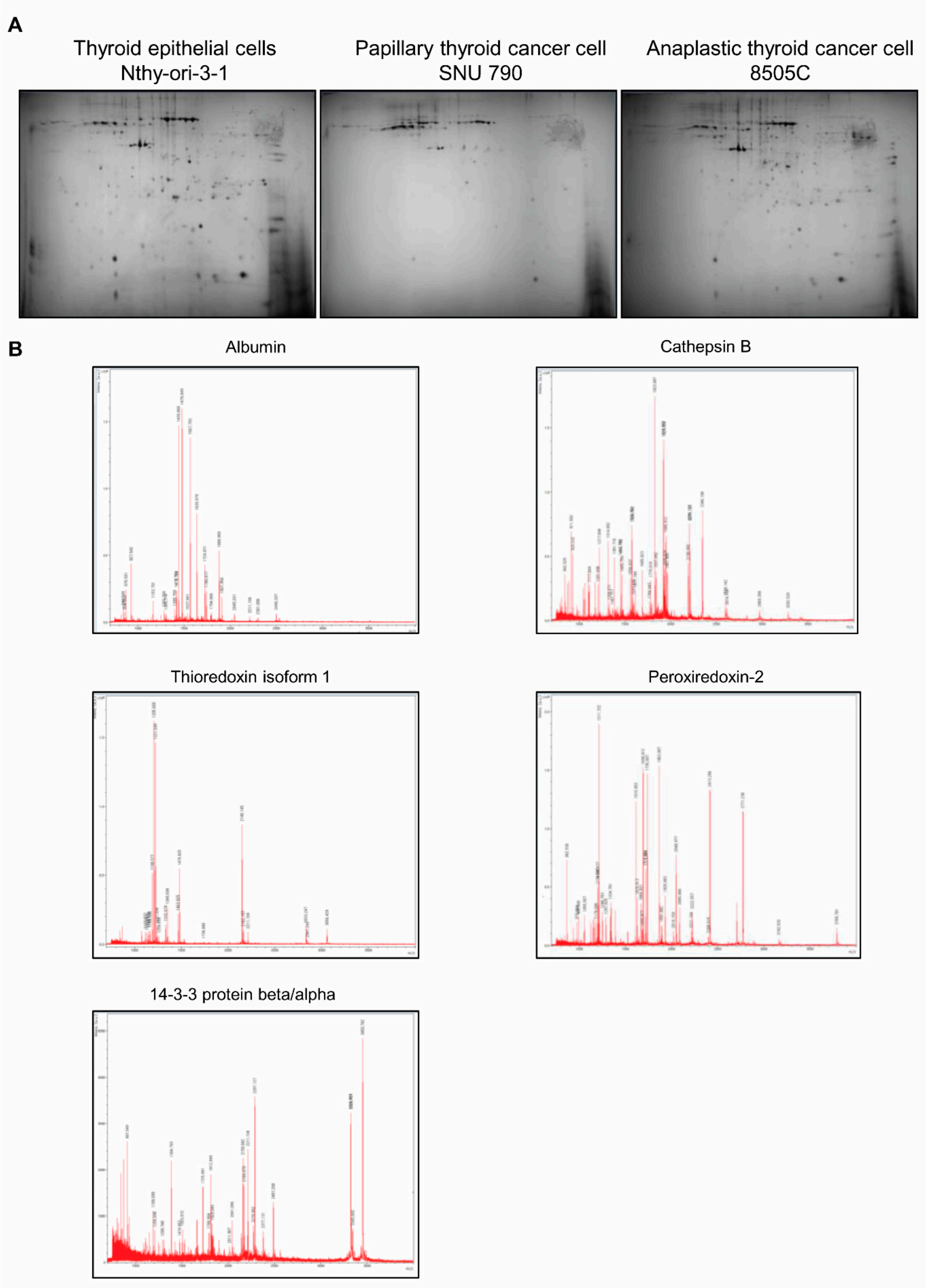

Figure S1. 2D-PAGE and MS analysis. **(A)** 2D-gel electrophoresis from conditioned medium of thyroid cancer cells. **(B)** The spectrum of the MALDI MS peptide map of identified proteins
